# Using Qualitative Disease Risk Analysis for Herpetofauna Conservation Translocations Transgressing Ecological and Geographical Barriers

**DOI:** 10.1007/s10393-015-1086-4

**Published:** 2015-12-22

**Authors:** Mariana Bobadilla Suarez, John G. Ewen, Jim J. Groombridge, K. Beckmann, J. Shotton, N. Masters, T. Hopkins, Anthony W. Sainsbury

**Affiliations:** 1 Institute of Zoology, Zoological Society of London, Regent’s Park, London, NW1 4RY UK; 2 Durrell Institute of Conservation and Ecology, University of Kent, Giles Lane, Canterbury, CT2 7NZ UK

**Keywords:** Translocations, Reintroductions, Reptiles, Amphibians, Disease management, Conservation

## Abstract

Through the exploration of disease risk analysis methods employed for four different UK herpetofauna translocations, we illustrate how disease hazards can be identified, and how the risk of disease can be analysed. Where ecological or geographical barriers between source and destination sites exist, parasite populations are likely to differ in identity or strain between the two sites, elevating the risk from disease and increasing the number and category of hazards requiring analysis. Simplification of the translocation pathway through the avoidance of these barriers reduces the risk from disease. The disease risk analysis tool is intended to aid conservation practitioners in decision making relating to disease hazards prior to implementation of a translocation.

## Introduction

The types of translocations employed for wildlife conservation include reintroduction, population reinforcement, assisted colonisation and ecological replacement (Seddon et al. 2014). Translocations have become increasingly commonplace in conservation, with an increasing variety of taxonomic groups being moved (Seddon et al. 2005) including within the herpetofauna (Germano and Bishop 2009; Ewen et al. 2014). Recovery efforts for many amphibian species have been reliant on translocation as a recovery tool (Griffiths and Pavajeau 2008). One concern associated with wildlife translocations is that the released individuals, or other individuals within the wider destination ecosystem, may suffer from disease linked to the translocation process. This may be a particular concern in amphibians, where close to 25 % of all extinct and threatened species on the IUCN Red List cite disease as a possible cause of decline (Heard et al. 2011) High-profile emerging infectious diseases that have impacted free-living herpetofauna include, *Batrachochytrium dendrobatidis* (Bd)-associated disease and *Batrachochytrium salamandrivorans* (Bsal)-associated disease, ranaviral disease and Snake Fungal Disease (SFD) (Pounds et al. 2006; Teacher et al. 2010; Allender et al. 2011; Miller and Gray 2010; USGS 2013; Hyatt et al. 2002; Martel et al. 2013). Therefore, in undertaking translocations of herpetofauna, as in any other taxonomic group, it is crucial to assess and manage risk from disease.

Parasites may cause disease in released animals resulting in establishment failure (a failed translocation) or, if novel to the release site, may cause other species at the release site to decline through disease. In this manuscript, we define parasites as infectious agents including viruses, bacteria, fungi, protozoa, helminths and ectoparasites. Parasites that are novel and introduced to release sites are a type of alien species (sensu Blackburn and Ewen submitted this volume). Release of animals may also alter the transmission dynamics of endemic parasites at the destination site (due to host aggregation) and increase the probability of an infectious disease outbreak (Aiello et al. 2014). At its heart, therefore, the occurrence of disease in a translocation often relates to differing host–parasite communities between source and destination locations, potentially influenced by stressors acting on the individuals moved (Sainsbury and Vaughan-Higgins 2012). For example, the reintroduction of Mallorcan midwife toads (*Alytes muletensis*) reared in captive breeding facility is likely to have accidentally co-introduced an alien parasite, Bd, to the recipient environment which was associated with disease outbreaks in native Mallorcan amphibian populations (Walker et al. 2008). Furthermore, the critically endangered mountain chicken frog (*Leptodactylus fallax*) reintroduction programme in the island nation of Montserrat has been hindered by the continued presence of *Bd* during the reintroduction (Adams et al. 2014). Empirical evidence from across taxonomic groups demonstrates that as a consequence of translocations, alien parasites have caused major epidemics, with adverse effects at the ecosystem level (Sainsbury and Vaughan-Higgins 2012; Viggers et al. 1993; Dobson and Foufopoulos 2001).

In conservation translocations, the risks of disease from some select parasites may be known and management measures can be employed to mitigate them. However, the geographical distribution and/or pathogenicity of other parasites may not be apparent until after the translocation (Sainsbury and Vaughan-Higgins 2012; Ewen et al. 2012). Additionally, some parasites may be unknown or unidentified. There is a range of tools available to help identify infectious and non-infectious health hazards and to assess their level of risk from disease against various objectives (e.g. risk to the individuals being moved, to populations of the species at the destination sites and to the wider ecosystem including environmental and human health). Collectively, these tools are components of disease risk analysis (DRA). However, it remains unclear how widely these DRA methods are applied to conservation translocations. Although DRA guidelines have been published for wild animal translocations since 1992 (Davidson and Nettles 1992; De With et al. 1998; Corn and Nettles 2001; Neimanis and Leighton 2004; Hartley 2010), the literature has only recently started reporting how the methods have been applied to translocations for conservation purposes (e.g. Armstrong et al. 2003; Miller 2007; Hartley and Gill 2010; Sainsbury and Vaughan-Higgins 2012; Jakob-Hoff et al. 2014a) and how they may be integrated more closely with decision analysis (Ewen et al. 2015). We are unaware of any peer-reviewed publication of DRA application in the herpetofauna, a knowledge gap which may in part be due to a lack of guidance on implementation. Therefore, our focus here is to show how DRA has been applied in real case studies to provide worked examples to biodiversity managers.

Here we will briefly describe the qualitative disease risk analysis (DRA) method developed by Sainsbury and Vaughan-Higgins (2012) for conservation translocations. Our intention is not to review DRA methods and their development, but rather present a series of four case studies to show managers how these tools have been applied in translocations of herpetofauna in the UK. We focus particularly on defining the translocation pathway and explore how increasing the number of geographical and ecological barriers crossed on this pathway increases the complexity of risk. The four case studies include: the smooth snake (*Coronella austriaca*), the common European adder (*Vipera berus*) (hereafter: the adder), the pool frog *(Pelophylax lessonae)* and the sand lizard *(Lacerta agilis).* Each case study faced unique challenges, in particular, there are interesting contrasts between the influence of ecological and/or geographical barriers within the actual or proposed (as in the case of the adder) translocation pathway.

## Qualitative Disease Risk Analysis

The importance of a qualitative DRA lies in endeavouring to tackle the problem of infectious and non-infectious agent hazards in translocations in the face of uncertainty, including the scarcity of baseline data on the number, identity, pathogenicity and geographical distribution of parasite hazards (Sainsbury et al. 2012). To ensure appropriate judgement of risk is made by decision makers, it is important that a DRA is undertaken transparently (e.g. it must explicitly state any assumptions made due to gaps in knowledge).

The Sainsbury and Vaughan-Higgins (2012) method of conducting a DRA has been developed from previous qualitative DRA methods for wildlife (Davidson and Nettles 1992; Leighton 2002) and domestic animals (Murray et al. 2004). The DRA process described here involves completing a series of steps that follow a similar structure to the World Organization for Animal Health (OIE) guidelines for DRA in domestic animal movements between countries (Murray et al. 2004) but are tailored to the needs of wildlife translocations as described by Sainsbury and Vaughan-Higgins (2012). These steps consist of: (1) mapping out the translocation pathway, (2) hazard identification, (3) risk assessment, (4) risk management and (5) risk communication.

### Translocation Pathway

**Figure. 1:**
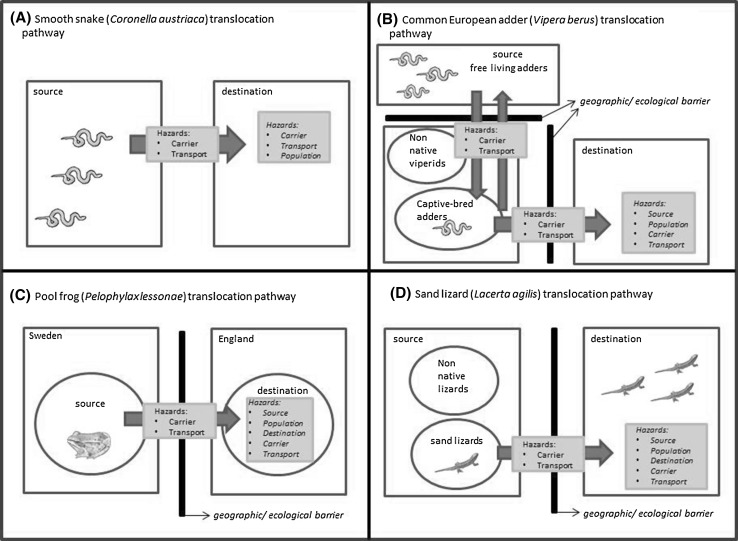
Translocation pathways. **a** The smooth snake translocation pathway included the collection of individuals over the course of several seasons from five to eight different sites. Smooth snakes were then moved to sites with assumed connectivity (i.e. no geographical or ecological barriers) at which the species was deemed locally extinct but was present historically. **b** The adder translocation pathway began with the initial capture of free-living female adders for captive breeding purposes, followed by the capture of males for captive breeding. It was intended that juvenile adders either be returned to sites where they were present, reintroduced to sites where historical records of adders existed and where there was suitable habitat, or introduced to similar sites outside the study area but with suitable habitat. **c** The pool frog translocation pathway involved moving individuals from wild populations in Sweden to the UK taking into consideration the geographical barriers. **d** The sand lizard reintroduction pathway used captive-bred stock for reintroduction into historical sand lizard habitat. *Blue arrows* represent movement of individuals rather than movement of hazards. Hazards in *blue boxes* are placed on the segments of the translocation pathway where they would have an effect.

A translocation pathway is a visual representation of the route of the translocated animals that illustrates the points at which different types of hazards may potentially harm translocated individuals or the recipient ecosystem (Fig. 1a–d). Hazards can either be infectious or non-infectious. Infectious hazards are parasites that are either known to be associated with disease in the translocated species or species at the destination, and/or are novel to the destination, and/or are novel to the translocated species. Non-infectious hazards include nutritional deficiencies, toxins or objects capable of inflicting traumatic injury. We will largely focus on infectious hazards and their classification within the DRA method.

Infectious agents can be categorised into one or more hazard types according to the stage of the translocation pathway at which they act, and/or their novelty to the host’s immune system, and are termed as source, destination, transport, carrier, population or zoonotic hazards (see Table 1 for hazard definitions). Understanding which of these hazards, or combination of hazards, may be operating in a given translocation pathway allows practitioners to more clearly see why a hazard has been identified and enables them to consider either alternative translocation pathways in order to avoid a high risk of disease or to implement actions to manage those disease risks.

**Table 1:** Hazard Types and Definitions According to Sainsbury and Vaughan-Higgins (2012) and Masters and Sainsbury (2011).

Hazard type	Definition
Source hazard	The infectious agents or strains of these agents, carried by translocated individuals which are novel (alien) to the release environment (Sainsbury and Vaughan-Higgins 2012)
Destination hazard	The infectious agents found at the release environment to which the translocated animals are naïve (Sainsbury and Vaughan-Higgins 2012)
Carrier hazard	Those commensal organisms that cause disease when stressors reduce host immunocompetence and alter the host–parasite relationship (Sainsbury and Vaughan-Higgins 2012)
Transport hazard	Those hazards that may be encountered during the transport (between the source and destination) which are novel to the translocated animals and/or the release environment (Sainsbury and Vaughan-Higgins 2012)
Population hazard	Those non-infectious and infectious agents present at the release site that could potentially have a negative impact on a population as a whole but which are not necessarily novel to them (Sainsbury and Vaughan-Higgins 2012)
Zoonotic hazard	The infectious agents carried by the translocated species which can be transmitted to humans and potentially harm the latter (Masters and Sainsbury 2011)

A major consideration in a given translocation pathway is whether any geographical or ecological barriers are to be crossed. ‘Geographical barriers’ are natural and environmental barriers that prevent natural movement between populations (e.g. rivers, mountain ranges, seas). ‘Ecological barriers’ are those characteristics (for example: physical, behavioural, reproductive) that prevent interaction between populations, in the absence of geographical barriers, e.g. the populations may occupy different ecological niches. If either geographical or ecological barriers are crossed, source and destination hazards come into play, and the probability that translocated or recipient populations are exposed to novel parasites is increased. The distinction between a translocation pathway with and without geographical and ecological barriers is crucial, because empirical evidence shows that the major epidemics of disease associated with translocations have primarily arisen from source hazards (Sainsbury and Vaughan-Higgins 2012; Cunningham 1996; Dobson and Foufopoulos 2001). An assumption that there is a high probability that such alien (source and destination) hazards are absent or minimal in a given translocation gives the translocation manager confidence that the overall risk from disease of a given translocation is markedly reduced. Therefore, if source and destination environments are not separated by barriers, source and destination hazards do not require consideration and overall risk from disease is reduced.

### Hazard Identification

Through literature review, elicitation of expert opinion from ecologists, epidemiologists and pathologists, and/or screening of populations, infectious and non-infectious agents are identified that have potential to cause disease through novelty, and/or known pathogenicity, to the individuals that are translocated, and/or to the populations of the same or closely related species at the destination, and/or potentially to the wider ecosystem. To be defined as a population hazard, there must be evidence that a parasite or non-infectious agent has an effect on population numbers and is capable of causing the population to decline, with an understanding that translocated populations may be small and more vulnerable to impacts from diseases. Searches in the literature can include diseases and/or parasites in the translocated species and closely related species. As each hazard is identified, it must also be classified into a hazard type (see step 1) and this classification justified using evidence to ensure a transparent process. Where parasites are of similar taxonomy and/or epidemiological parameters, they can be identified as a group, for example ‘gram-negative bacteria’. Searches in the literature and use of expert opinion enable an evaluation of the geographical and ecological barriers crossed in the translocation pathway and therefore enable the hazard type to be defined.

### Risk Assessment

This component involves four steps, namely (1) release assessment, (2) exposure assessment, (3) consequence assessment, and (4) risk estimation (Murray et al. 2004). The release assessment explains the pathway through which a translocated animal could be exposed and infected, or contaminated with the hazard and estimates the probability of infection in translocated individuals. It is important to note that for destination and population hazards, the release assessment does not apply as these hazards are, by definition, already present at the destination. The exposure assessment explains (1) how individuals of the same or closely related species at the destination could be exposed to and infected, or contaminated, with the hazard (source, transport, carrier hazards), (2) the probability of this exposure and infection occurring and (3) the probability of dissemination of the hazard through populations at the destination. For destination and population hazards, the exposure assessment explains how the translocated animals would become exposed to and infected with the hazard, and the probability of both this exposure and infection, and also dissemination occurring through populations at the destination. In the case of non-infectious agents, which can only be population hazards, the exposure assessment explains how the translocated animals would be exposed and the probability of occurrence of this exposure.

The consequence assessment then determines the consequences of exposure, and the probability that these will occur, contrary to the objectives that the translocation attempts to fulfil. The objectives against which consequences are estimated will most likely include consequences for the individuals moved, for the population of the same species if it is already present at the destination, and for the wider ecosystem at the destination. A zoonotic hazard can be additionally designated to any of the other hazard types and is an occupational health hazard. Finally, risk estimation integrates the results from all three assessments described above to provide an overall combined measure of the risks of disease that each hazard poses (Murray et al. 2004). These estimates will be influenced by the information available, values (e.g. risk attitude) of the specialist(s) undertaking the DRA and can vary from negligible risk to very high risk (Table 2). Guidelines for assigning these estimates are difficult to generalise and tend to change on a case by case basis. Overall, a reasoned, informed and transparent discussion of the risks of disease from each hazard is made and included within the DRA to justify each risk probability.

**Table 2:** Terminology Used to Describe the Likelihood or Probability Estimates When Undertaking a Disease Risk Analysis (Adapted From Murray et al. 2004).

Term	Definition
Negligible	Not worth considering, insignificant
Low	Less than average, coming below the normal level
Medium	Average, the usual amount, extent or rate
High	Extending above the normal level

### Risk Management

Risk management consists of identifying and evaluating management actions that may reduce the risks of diseases identified in previous steps. The need to identify management options is only required for those hazards deemed to be above negligible risk within any given translocation project. These decisions are normally made by the DRA specialist and project managers. Here, any specific management actions proposed may also include a review and monitoring component to improve the project teams’ knowledge of disease-related hazards and their level of risk (Murray et al. 2004).

### Risk Communication

Finally, risk communication is essential to ensure that decision makers have clear and transparent justification for each hazard’s risk status and risk management options. At the start of a DRA, a meeting is held with stakeholders to explain and discuss the DRA process and to agree a description of the translocation pathway. Further meetings may be held to discuss results as they become available. A written report is presented to the decision maker and stakeholders (those involved in the translocation planning and those affected by the translocation). The report clearly presents each of the preceding steps and provides all the information required for decision makers to make choices about how and whether a translocation should proceed. The DRA document should not tell the stakeholders what to do, but rather elucidate the options available to the managers, and the risk from disease presented by these options such that costs and benefits of the translocation can be considered alongside other evaluations, prior to proceeding. These management options and their associated risks are essentially dependent on the species being translocated, the translocation pathway and the quality, quantity and breadth of information available to construct and analyse them. In the following case studies, we omit the *risk communication* section as it simply summarises the previous four steps of the DRA process.

The following four case studies all use the above DRA method. Here we will present the details of each using the steps outlined above, while highlighting the major difficulties faced with each one. We will also describe how these difficulties were managed within the DRA, how they influenced the recommendations made and how the experience of conducting a DRA in one species can help inform its application to other species.

## Qualitative Disease Risk Analysis in Practice

### Crossing No Ecological or Geographical Barriers: Smooth Snakes (*Coronella austriaca*)

In 2010, a proposal was made to undertake a wild-to-wild translocation of smooth snakes to restock a dwindling remnant population in West Sussex from a nearby and more abundant source in Dorset.

#### Translocation Pathway

It was believed that the translocation pathway for smooth snakes did not cross barriers (Fig. 1a) because the individuals were being taken from a source area with assumed connectivity of parasite assemblages through sympatric reptiles to their destination. This inference was made by the specialist wildlife veterinarians who completed the DRA in consultation with expert reptile ecologists.

#### Hazard Identification

Parasites known to be present in smooth snakes, and other ophidian species, were identified following a detailed review of the published literature (found using keywords ‘infectious disease’ and ‘snakes’) and reptile disease and medicine textbooks, then evaluated for hazard type and their inclusion as hazards justified. Table 2 shows the list of pathogenic agents (infectious and non-infectious) evaluated and those which were identified as hazards (Table 3). No source or destination hazards were identified because the translocation pathway was not believed to cross geographical or ecological barriers.

**Table 3:** Potential Infectious and Non-infectious Pathogenic Agents Considered for Inclusion in the Smooth Snake DRA as Hazards (Reproduction Permission Masters and Sainsbury 2011).

Potential hazard	Type of Parasite	Hazard type
Ophidian Paramyxovirus (OPMV)	Virus	Carrier and transport
Adenoviruses	Virus	Carrier and transport
Iridoviruses (Snake Erythrocyte Virus (SEV) and ranavirus)	Virus	Carrier and transport
Reoviruses	Virus	Carrier and transport
Other viruses (Inclusion Body Disease virus retrovirus (IBD), herpesviruses, parvoviruses, retroviruses (other than IBD), caliciviruses, picornaviruses and ‘arboviruses’ including flaviviruses and togaviruses)	Virus	Not a hazard
Gram-negative bacteria (including *Pseudomonas* sp., *Aeromonas* sp., *Klebsiella* sp., *Proteus* sp., *Eschericia coli, Citrobacter* sp., *Acinetobacter* sp., *Enterobacter* sp., *Flavobacter* sp., *Providencia* sp., *Serratia* sp., *Morganella* sp., *Salmonella* sp.)	Bacteria	Carrier and transport
*Salmonella* sp.	Bacterium	Zoonotic
Mycobacteria other than *Mycobacterium* *tuberculosis* complex (MOTT) (especially *M. marinum*)	Bacterium	Destination and zoonotic
*Chlamydia* sp. (especially *C. pneumoniae*)	Bacterium	Carrier, transport and zoonotic
*Coxiella burnetii*	Bacterium	Carrier and zoonotic
Other Bacteria (*Leptospira sp*., and *Mycoplasma sp*.)	Bacteria	Not a hazard
*Chrysosporium* anamorph of *Nannizziopsis vriesii* (CANV)	Fungus	Transport and zoonotic
*Aspergillus* sp., *Penicillium* sp., *Paecilomyces* sp., *Fusarium* sp.	Fungi	Destination
*Candida* sp.	Fungus	Carrier
Other fungi (*Cryptococcus* sp., *Trichosporon* sp., Dermatophytes and Microsporidia)	Fungi	Not a hazard
*Cryptosporidium serpentis*	Protozoa	Carrier and transport
*Entamoeba invadens*	Protozoa	Transport
Other protozoa (*Eimeria* sp., *Sarcocystis* sp., Flagellates, *Plasmodium* sp. and *Haemoproteus* sp.)	Protozoa	Not a hazard
Haemogregarines (especially *Hepatozoon* sp.)	Haemoparasite	Carrier
Nematodes of Ascaroidea superfamily, Diaphanocephaloidea superfamily (especially *Kalicephalus* sp.), Rhabitoidea superfamily (especially *Rhabdius* sp. and *Strongyloides* sp.) and Acanthocephala	Helminths	Carrier
Other Helminths [Cestodes (Orders Pseudophyllidae, Proteocephalidae, Cyclophyllidae), Trematodes (Digenetic families Ocheotosomatidae and Plagiorchiidae, and the family Dipostomatidae) and Nematodes (e.g. Filaroidea superfamily)]	Helminths	Not a hazard
Pentastomids	Crustacea	Transport and carrier and zoonotic
Mites (especially *Ophionyssus natricis*)	Ectoparasites	Carrier and transport
Other ectoparasites (Acari and Diptera)	Ectoparasites	Not a hazard
Agricultural chemicals	Toxin	Not a hazard

#### Risk Assessment

Of the disease hazards identified in the DRA, the viruses identified were generally found to be the highest risk hazards, but only when evaluated as transport hazards. These high-risk hazards included ophidian paramyxovirus, adenoviruses, iridoviruses and reoviruses. In general, these hazards were deemed of high risk because of the likelihood that the smooth snakes could be exposed to alien strains of these viruses, through direct or indirect contact with exotic snakes, *en route* to their destination, and the high probability that they could then transmit these exotic strains to naïve animals at the destination. The other high-risk hazard was *Salmonella* spp., a carrier, transport, and zoonotic hazard (also included in the gram-negative bacteria hazard grouping) (Table 3).

#### Risk Management

Three general management measures were proposed, which included (a) biosecurity: in order to reduce the probability of infection of smooth snakes with any novel infectious agent during the translocation (e.g. reducing the risk of disease from transport hazards through the establishment of quarantine barriers at every stage of the translocation, for example by designating a specific quarantine zone within the transporting vehicle in which only smooth-snake-dedicated tools and equipment, such as vivaria, were used), (b) husbandry: in order to reduce the probability for any stress-induced immuno-suppressive effect that may precipitate disease in smooth snakes being translocated, for example in association with carrier hazards and (c) occupational health measures: in order to reduce the probability of the transmission of zoonotic infectious agents from translocated smooth snakes to people working on the translocation. As a consequence of the perceived absence of source and destination hazards in a translocation which does not cross barriers, Masters and Sainsbury (2011) believed they could limit disease risk management to these restricted measures.

### Potential Exposure to Alien Parasites: The Adder (*Vipera berus*)

The adder is the UK’s only venomous snake, and has historically been persecuted because of its perceived threat to humans and their animals (Beebee and Griffiths 2000; Prestt 1971). Currently, the suspected main threat to the adder’s persistence is habitat fragmentation and degradation (JNCC 2010). In 2010, a reintroduction project was proposed for supplementing adder populations in west-central England. The proposal involved the release of captive-bred offspring of wild-caught adults, which had been captured and taken into captivity prior to the DRA being conducted. These individuals were housed within a zoo which possessed exotic species of wide geographical origin including non-native vipers. It was the intention to release the progeny of these adders into the same forest whence the breeding adults had been collected.

#### Translocation Pathway

It was assessed that the proposed translocation pathway included the transfer of the adders across ecological and geographical barriers because there was potential contact and transmission of parasites between non-native vipers and the adders in the zoological collection (Fig. 1b) (Beckmann et al. 2014). By breeding adders in the zoo setting, project managers had inadvertently created the conditions for parasites to cross geographical and ecological barriers and hence there was a risk of disease from source hazards.

#### Hazard Identification

A list of the non-native reptiles in the zoological collection was used to identify a list of potential infectious agent hazards in conjunction with a review of the scientific literature and expert opinion (Beckmann et al. 2014) (Table 4). Source hazards were included because of the potential for pathogens to cross geographical and ecological barriers during the translocation pathway. No destination hazards were included because at the time the DRA was conducted; it was assumed that there would be no geographical or ecological barriers between the source of the wild-caught breeding adults and the destination of their progeny which were to be released in the same forest from which the adults were taken. It was assumed that the progeny would be exposed to parasites present in this forest through contact with the breeding adults.

**Table 4:** Infectious Hazards Identified Through DRA for Common European Adder Translocation (Reproduction Permission Beckmann et al. 2014).

Infectious hazard	Type of parasite	Hazard type
Reptilian paramyxovirus (PMV)	Virus	Source
Adenoviruses	Virus	Carrier
Reoviruses	Virus	Source
Iridoviruses	Virus	Source
Gram-negative bacteria	Bacteria	Carrier and zoonotic
Mycobacteria other than *Mycobacterium* *tuberculosis* complex	Bacterium	Population and zoonotic
*Chlamydia* sp.	Bacterium	Source and zoonotic
*Aspergillus* sp., *Penicillium* sp., *Paecilomyces* sp., *Fusarium* sp.	Fungi	Carrier
*Candida* sp.	Fungus	Carrier
*Entamoeba invadens*	Protozoa	Source
Coccidia (including *Cryptosporidium serpentis*)	Protozoa	Source and carrier
Haemogregarines	Protozoa	Source and carrier
Helminths (including nematodes and acanthocephalans)	Helminth	Source and carrier
Pentastomids	Crustacea	Source and zoonotic
Mites (especially *Ophionyssus natricis*)	Ectoparasite	Source and carrier

#### Risk Assessment

Of fifteen parasites, or parasite groups, identified as hazards two source hazards (*Entamoeba invadens* and reoviruses*)* were estimated to present a high risk to the reintroduction. Both of these hazards were considered to present a high risk, since there was potential for captive-bred adders to become infected as a result of indirect transmission from exotic reptiles, and for the parasites to be released (and even to cause disease) in free-living adder populations at the release site (Beckmann et al. 2014). *Entamoeba invadens*, a protozoan parasite, had not been documented in the reptile species housed in the zoo, or in free-living adders, yet it was known to be a commensal parasite of captive reptiles (Barnard and Upton 1994; Wilson and Carpenter 1996). Reoviruses were also deemed high risk, in light of their ability to “jump” to other species. In fact, two wild-caught adult adders in the captive breeding programme at the zoological collection died and tested positive for reovirus at post-mortem examination; the pathological significance of the infection was unclear in each case. The origin of reovirus infection was uncertain, and in the absence of data regarding the presence/prevalence of reovirus in wild adders and sympatric reptiles in the UK, there was concern that the infection may have originated from captive exotic reptile species and may therefore be alien to native herpetofauna (Beckmann et al. 2014).

#### Risk Management

The DRA report proposed two alternative translocation strategies as a means to reduce the risk from disease for the adder, namely (1) a direct wild-to-wild translocation, or (2) establishment of a dedicated captive breeding facility at the destination site (rather than within the zoological collection). These alternatives were predicted to greatly reduce the ecological and geographical barriers in the translocation pathway (Beckmann et al. 2014) and therefore reduce source and destination hazards. Both these approaches are currently under consideration. In preparing for a future wild-to-wild translocation, analysis of the literature (Shotton and Sainsbury 2014) showed that it cannot be assumed that adder populations within England are contiguous because (1) long-term population studies of adders have shown strong site philopatry and high hibernacula fidelity (Phelps 2004); (2) vipers show a low migration potential (Hand 2013); and (3) high genetic differentiation exists between adder populations in Europe (Durrant 2014; Ursenbacher et al. 2009) and therefore population structuring may reveal ecological barriers and hence conditions where source and destination hazards may be present.

### Crossing Geographical and Ecological Barriers: Pool Frogs (*Pelophylax lessonae*)

In the late 1990s, the northern clade pool frog became extinct in England (Beebee 2013; Beebee et al. 2005). Following extensive planning (Buckley and Foster 2005), reintroduction of wild-caught pool frogs collected from Sweden and transported to England occurred between 2005 and 2008 (Baker and Foster 2015). As this was one of the first translocations we were involved with, our method of DRA at that time was more rudimentary (Sainsbury et al., in press).

#### Translocation Pathway

This translocation crossed geographical and ecological barriers when moving pool frogs from mainland Sweden to an isolated landmass (England) (Fig. 1c), and therefore source and destination hazards were of greatest concern.

#### Hazard Identification

Hazard identification was achieved through detailed literature review and screening for parasites in the source population of pool frogs in Sweden and four native amphibian species at the destination site in England (Table 5). The literature review revealed a relative lack of information regarding parasites of amphibians in Sweden (Sainsbury et al., in press), and in the context of the global amphibian decline and its association with infectious disease, it was decided that it was important to obtain better information on parasites through screening of pool frogs in Sweden and amphibians at the reintroduction site in England. Cunningham et al. (2001) expressed concerns that “potentially catastrophic epidemic ranavirus disease or cutaneous chytridiomycosis” could be co-introduced with any translocation of amphibians, including pool frogs, and therefore an emphasis was placed on identifying the presence and absence of ranaviruses and Bd in Swedish pool frogs. Source and destination hazards were identified because geographical and ecological barriers were present between Sweden and England. The results of screening showed no ranaviruses or Bd in the Swedish pool frog populations sampled, but these agents were known to be present in England and therefore they were identified as destination hazards. Two protozoan parasites *Trypanosoma rotatorium* and unidentified intestinal opalinid cysts were detected in pool frogs from Sweden and identified as source hazards.

**Table 5:** Infectious Hazards for the Pool Frog DRA (Adapted From Sainsbury et al., in press).

Infectious hazard	Type of parasite	Hazard type
Ranaviruses	Virus	Destination
*Batrachochytrium dendrobatidis*	Fungus	Destination
*Amphibiocystidium ranae*	Mesomycetozoea	Destination
Unidentified intestinal protozoa	Protozoa	Destination
*Trypanosoma rotatorium*	Protozoa	Source
Unidentified intestinal Opalinid cysts	Protozoa	Source and transport

#### Risk Assessment

The DRA process in this case study focussed on source hazards (by estimating the probability of co-introduction and the likelihood of consequences based on pathogenic capabilities) and destination hazards (by estimating the probability of establishment of a parasite in the released population of pool frogs and the probability that an established parasite would be pathogenic). Two high-risk hazards were analysed: ranaviruses and Bd in England as destination hazards.

#### Risk Management

When the pool frogs were translocated, strict biosecurity was adopted to try to protect the small reintroduced pool frog population from these destination hazards until the pool frog population could become established. The disease risk management protocol included using amphibian-proof fencing at the release site to create a quarantine barrier to try to prevent ingress of destination hazards, health examinations of pool frogs before and after translocation, and pathological examination of any dead animals found (see Vaughan-Higgins et al. 2015, this volume).

### Captive Breeding in Multiple Locations: Sand Lizard Reintroduction (*Lacerta agilis*)

Translocations of sand lizards began in 1968 (Moulton et al. 2011) and were mostly carried out from wild-to-wild for mitigation purposes, driven by impending habitat disturbances due to development and without DRA. Beginning in the 1990s, the focus of conservation translocations for sand lizards shifted to a preference for reintroduction using captive-bred stock. A post hoc DRA was requested by Natural England in order to assess the risks from disease associated with this long-term captive breeding and release programme (Lloyd and Sainsbury 2003). It is important to note that several of the captive-breeders held collections which included exotic reptile species and which did not have biosecurity measures in place (Lloyd and Sainsbury 2003).

#### Translocation Pathway

The reintroduction of sand lizards into existing and historical habitats was carried out using captive-bred stock from several breeders (Fig. 1d). This pathway included the crossing of geographical and ecological barriers because non-native reptiles were present without biosecurity.

#### Hazard Identification

Similar to the adder scenario, special attention was placed on the possibility of direct and indirect contact with exotic reptile species that were in shared captive breeding facilities (Lloyd and Sainsbury 2003). Visits to breeders and/or surveys of their facilities were invaluable when further developing the DRA. A full list of hazards considered for this DRA can be found in Table 6.

**Table 6:** Infectious Hazards for the Sand Lizard DRA.

Infectious hazard	Type of parasite	Hazard type
Adenovirus	Virus	Source, carrier and transport
Herpesviruses	Virus	Not a hazard
Reovirus	Virus	Source, carrier and transport
Iridoviruses	Virus	Source, carrier and transport
Paramyxovirus (PMV)	Virus	Source, carrier and transport
Gram-negative bacteria *(Aeromonas* spp., *Corynebacterium* spp., *Klebsiella* spp., *Proteus* spp., *Pseudomonas* spp., Salmonella spp.)	Bacteria	Carrier and zoonotic
Mycobacteria	Bacteria	Zoonotic
Trichomonads	Protozoa	Transport and carrier
*Entamoeba invadens*	Protozoa	Source and transport
*Coccidia* (*Eimeria*, sp. *Isospora* sp. and *Cryptosporidia* sp.)	Protozoa	Source, transport and carrier
*Haemogregarina*, *Hepatozoon*, *Haemoproteus*, *Plasmodium*, *Trypanosomes*	Haemoparasites	Source and carrier
Non-native nematodes *Mesocestoides* spp., *Oswaldocruzia filiformis*, *Metaplagiorchis molini*, *Oochoristica tuberculata*	Helminths	Source
Non-native cestodes	Helminths	Source
Non-native trematodes	Helminths	Source
Pentastomids	Crustacea	Source and zoonotic
*Ophionyssus saurarum*, *Ixodes ricinus*, *Uropoda* sp.	Ectoparasites	Destination

#### Risk Assessment

The DRA found iridoviruses, paramyxoviruses, *Entamoeba invadens* and mycobacteria to be of highest risk, mainly because of evidence suggesting catastrophic consequences through epidemic disease, should these parasites be released into the destination ecosystem as novel agents (contracted from exotic reptiles).

#### Risk Management

In this case it was logistically difficult to call for a relocation of the captive breeding facility to the release site. Therefore, the captive breeding facilities were to be placed under permanent quarantine, which would allow for strict biosecurity practices to be established, including barrier methods to minimise exposure to non-native species and their parasites.

## Discussion

We have illustrated how DRA can help during the planning stages of translocations to better identify which infectious agents may be hazards and what options may be available to manage the risk of disease they might present. DRA is, in essence, a process for working with uncertainty in hazard identification and consequence assessment and making risk-sensitive management decisions based on this information (Ewen et al. 2015). Importantly, the method chosen in all of these case studies was made transparent by justifying which hazards were considered and why. Ideally, potential management actions are also supported by evidence. Whilst this approach does not mean risks are removed and all hazards are identified, it does provide a practical and rational approach to assessing disease-related risks.

Through the use of these four examples, we have shown that the more complicated a translocation pathway is (i.e. the more barriers involved), the more complicated the DRA process will be where barriers are crossed source and destination hazards must be analysed and the hazard list will be lengthened. Examples of complicated translocation pathways resulting in more complex DRAs can also be seen in the Eurasian crane (*Grus grus*) (Vaughan and Sainsbury 2010; Sainsbury and Vaughan-Higgins 2012) and short-haired bumblebee (*Bombus subterraneu*s) (Brown et al. this volume) reintroduction to England, Regent honeyeaters (*Xanthomyza Phrygia*) in Australia (Jakob-Hoff et al. 2014a), and Eastern wild turkeys (*Meleagris gallopavo silvestris*) to Canada (Neimanis and Leighton 2004). The most straightforward solution to reduce disease risks in these cases is simplification of the translocation pathway, for example by avoiding holding animals for translocation in multi-species, multi-origin captive facilities. Alternatives may be feasible with options to place captive breeding facilities at the release site. This method was successfully used for reintroduction of cirl buntings (*Emberiza cirlus*) in south west England (McGill et al. 2010). Special care must be taken to ensure that when trying to eliminate obvious geographical and ecological barriers, similar to those in the pool frog scenario, one also considers those obscure ecological barriers as identified in the adder scenario. Epidemiological principles also support a reduction in duration and distance of transport during a translocation, which likely also reduce stress. Transport and carrier hazards are more likely to be associated with disease when these transport durations are longer and more complex.

This DRA approach (Sainsbury and Vaughan-Higgins 2012) follows a reasoned, methodical and widely accepted set of guidelines (Jakob-Hoff et al. 2014b). Its most valuable asset is the ability to identify and evaluate those sometimes overlooked disease risks with transparency. In the face of increasingly apparent parasite threats to global biodiversity (Daszak et al. 2000), this tool can help to consider the risks from disease in translocation. While the challenges we face when compiling a DRA are many (e.g. often including a lack of information on parasite presence, identity, geographical distribution and virulence), further application and critical evaluation can help to continually improve our application of these tools.
